# Growth Repressor GmRAV Binds to the GmGA3ox Promoter to Negatively Regulate Plant Height Development in Soybean

**DOI:** 10.3390/ijms23031721

**Published:** 2022-02-02

**Authors:** Yongguo Xue, Yuntong Zhang, Jinming Shan, Yujia Ji, Xiaoming Zhang, Wenbin Li, Dongmei Li, Lin Zhao

**Affiliations:** Key Laboratory of Soybean Biology of Ministry of Education China, Northeast Agricultural University, Harbin 150030, China; xyg81@126.com (Y.X.); yuntong0104@163.com (Y.Z.); shanjinming1997@163.com (J.S.); mdjjiyujia@163.com (Y.J.); zhangxiaoming@neau.edu.cn (X.Z.); wenbinli@neau.edu.cn (W.L.)

**Keywords:** soybean, *GmRAV*, plant height, *GmGA3ox*, GA content

## Abstract

Plant height is an important component of plant architecture, and significantly affects crop quality and yield. A soybean *GmRAV* (Related to *ABI3/VP1*) transcription factor containing both AP2 and B3 domains is a growth repressor. Three *GmRAV-overexpressing* (*GmRAV-ox*) transgenic lines displayed extremely shorter height and shortened internodes compared with control plants, whereas transgenic inhibition of *GmRAV* expression resulted in increased plant height. *GmRAV-ox* soybean plants showed a low active gibberellin level and the dwarf phenotype could be rescued by treatment with exogenous GA_3_ treatment. ChIP (Chromatin immunoprecipitation)-qPCR assay showed that GmRAV could directly regulate the expression of the GA4 biosynthetic genes *GA3-oxidase* (*GmGA3ox*) by binding two CAACA motifs in the *GmGA3ox* promoter. The *GmGA3ox* promoter was bound by GmRAV, whose expression levels in leaves were both elevated in *GmRAV-i-3* and decreased in *GmRAV-ox-7* soybean plants. Transient expression assay in *N. benthamiana* also showed that the *proGmRAV:GmRAV-3F6H* effector strongly repressed the expression of LUC reporter gene driven by *GmGA3ox* promoter containing two CAACA motifs. Together, our results suggested that GmRAV protein repressed the expression of *GmGA3ox* by directly binding to the two CAACA motifs in the promoter to limit soybean plant height.

## 1. Introduction

Soybean (*Glycine max* (L.) Merr.) is an important crop for plant oil and protein and provides more than a quarter of the world protein for livestock feed and human consumption [1]. Soybean yield is affected by plant height, node number, internode length, branch number, and seed size, etc. [2]. Plant height is an important trait of plant ideotypes, and a relatively shorter stem length contributes to increasing yield in breeding programs [3,4]. In the 1960s, with the promotion of dwarf and semi-dwarf varieties in the ‘Green Revolution’, crop production sharply increased. Some of the Green Revolution genes have been cloned and used in crop improvement, such as *sd1* in rice [5] and *Rht1* in wheat [6]. A gibberellin 20-oxidase (GA20ox) encoded by *sd1* can reduce endogenous gibberellin (GA) levels and the reduced levels result in the short stature of rice variety IR8 [5,7]. However, only a few genes that influence plant height have been cloned in soybean. For example, *GmDW1*(*dwarf*) was an ent-kaurene synthase, and the *dw* mutant displayed plant height reduction and internode shortening in soybean [8]. Circadian clock gene *LATE ELONGATED HYPOCOTYL* (*LHY*) encodes the morning-expressed MYB transcription factor. The quadruple mutant of *GmLHY* displayed reduced plant height and shortened internodes in soybean [9].

The RAV family members contain a B3 domain and an AP2 domain. Therefore, the members of this family can be classified as members of B3 superfamily or AP2/EREBP family [10]. The B3 domain is a DNA-binding domain, which consists of about 110 amino acids, forming a β-sheets and two α-helix [11,12]. The AP2 domain as a DNA-binding domain was first identified in Arabidopsis, which consisted of 57–66 amino acids [13,14]. This family played various roles in plant processes, such as flowering development, bud outgrowth, leaf senescence, hormone signaling, and stress responses [15,16,17,18,19]. Previously, *GmRAV-overexpressing* transgenic plants displayed later flowering time, shorter height and fewer numbers of leaves. *GmRAV-RNAi* transgenic lines showed the opposite phenotype, which suggested *GmRAV* played important roles in flowering time, plant height and leaf number in soybean [19]. *GmRAV* delayed soybean growth period by repressing the expression of florigen homolog *GmFT5a* [19].

In *Arabidopsis thaliana* and rice as model plant species, it has been reported that GAs is one of the most important phytohormones that influence plant height. For example, mutations of *GA3-oxidase* (*GA3ox*) genes could cause the dwarf phenotypes in Arabidopsis and rice [20,21]. However, the molecular mechanism of the *GmRAV* gene involved in the regulation of soybean height has not been characterized. In this study, we investigated the functions of *GmRAV* in soybean plant height, using overexpression and knockdown transgenic soybean lines to carry out expression analyses. We found that the dwarf phenotypes of *GmRAV-ox* soybean plants with low active gibberellin levels could be rescued by treatment with exogenous GA_3_ treatment. GmRAV could directly regulate the expression of *GmGA3ox* by binding two CAACA motifs in the *GmGA3ox* promoter. GmRAV protein repressed the expression of *GmGA3ox* to reduce gibberellin levels to limit soybean plant height. Our results demonstrated that GmRAV was involved in the regulation of plant height directly by mediating the key components of the GA synthesis pathway.

## 2. Results

### 2.1. GmRAV Inhibited Plant Height and Yield in Soybean

**Figure 1:**
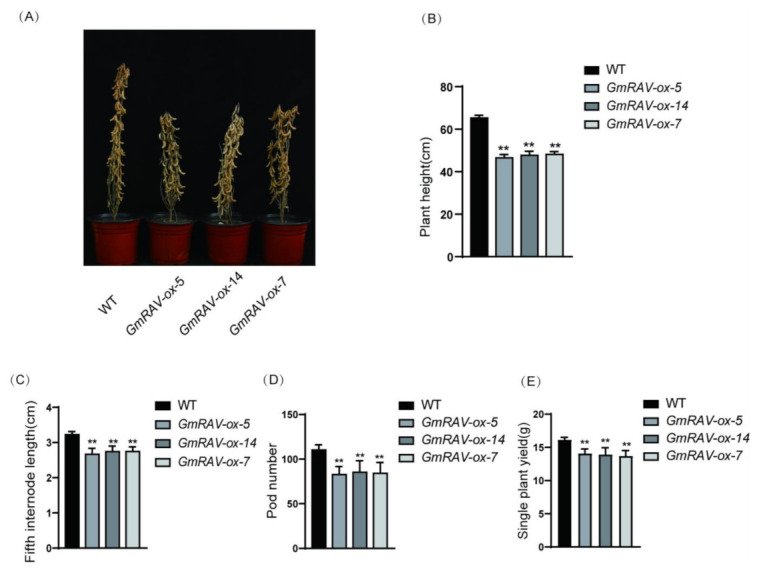
Phenotypes and agronomic traits of three T_4_ generation transgenic lines *GmRAV-ox-5*, *GmRAV-ox-14* and *GmRAV-ox-7* plants. (**A**) Phenotypes of three T_4_ generation transgenic lines at maturity stage. (**B**) Plant height at maturity stage. (**C**) The fifth internode length at maturity stage counted from the top. (**D**) Pod number per plant. (**E**) Single plant yield per plant. A total of 15 plants were scored for each line and WT. Values are shown as means ± standard deviation (SD) (*n* = 15). Student’s *t*-test, ** *p* < 0.01.

**Figure 2:**
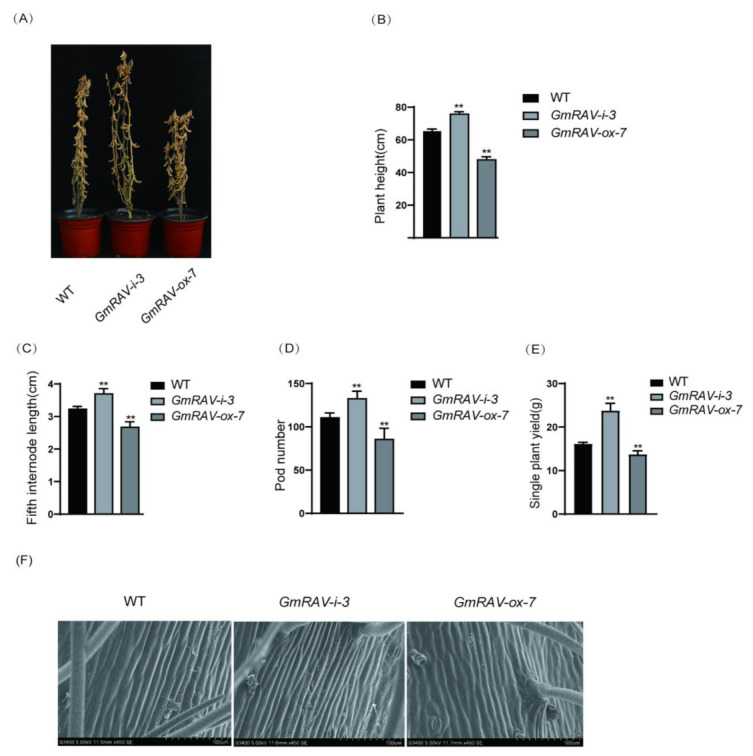
Phenotypes and agronomic traits of T_7_ generation *GmRAV-i-3* and T_4_ generation *GmRAV-ox-7* plants. (**A**) Phenotypes of soybean wild type, *GmRAV-i-3* and *GmRAV-ox-7* plants at maturity stage. (**B**) Plant height at maturity stage. (**C**) The fifth internode length at maturity stage counted from the top. (**D**) Pod number per plant. (**E**) Single plant yield per plant. (**F**) Cellular size analysis of WT, *GmRAV-i-3* and *GmRAV-ox-7* soybeans. Scanning electron microscope images of internode epidermal cells of WT, *GmRAV-i-3* and *GmRAV-ox-7* plants. Scale bars, 100 µm. A total of 15 plants were scored for each line and WT. Values are shown as means ± SD (*n* = 15). Student’s *t*-test, ** *p* < 0.01.

Three T_4_ generation transgenic lines *GmRAV-ox-5*, *GmRAV-ox-7* and *GmRAV-ox-14* plants showed shorter height due to the shorter internode lengths without the change of internode numbers, which were similar to GA-deficient mutants in phenotypes compared with control plants (Figure 1A–C). The final height of the mature plant was smaller compared to WT. *GmRAV-ox* also showed reduced pod numbers and single plant yield (Figure 1D,E). In contrast, T_7_ generation transgenic *GmRAV-i-3* soybean displayed increased plant heights due to the larger internode lengths, as well as increased pod numbers and single plant yield (Figure 2A–E). To investigate the cellular basis of the extension in the length of stem internodes, epidermal cells on the stem were examined by scanning electron microscopy (SEM). The epidermal cells in the stem internodes of the *GmRAV-ox-7* plants were smaller than those in WT plants in longitudinal direction (Figure 2F), which implied that the dwarf phenotype of *GmRAV-ox-7* plant was mainly caused by a reduced cell size. The dwarf and late-flowering phenotypes of *GmRAV-ox* plant were similar to that of GA-deficient and GA-insensitive mutants.

### 2.2. Dwarfism of GmRAV-ox Soybean Rescued by Exogenous GA_3_

**Figure 3:**
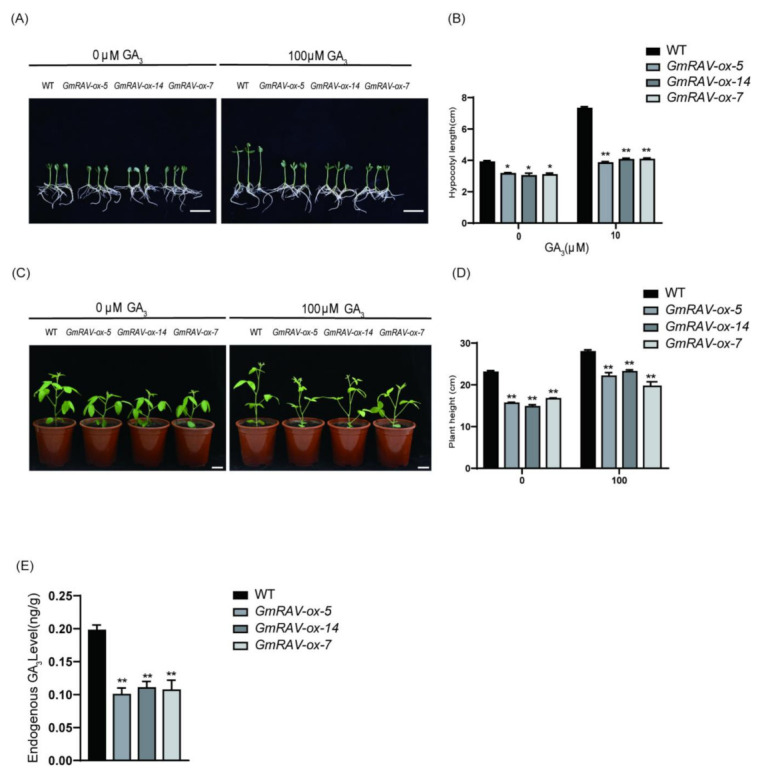
The restoration of hypocotyl length and plant height of three transgenic *GmRAV-ox* lines by exogenous addition of GA_3_. (**A**,**B**) Hypocotyl lengths of 8-day-old soybeans in response to 0 and 10 µM GA_3_. Hypocotyl length was measured using ImageJ software. At least 10 seedlings per treatment were checked. For each experiment, three technical replicates were conducted. (**C**,**D**) Plant height lengths of 26-day-old soybean supplemented with/without 100 µM GA_3_ treatments. (**E**) Measurement of endogenous GA_3_ levels in the leaves of 20-day-old soybean. Ten plants were analyzed for each line each time and the experiments were repeated three times. Values are shown as means ± SD (*n* = 10). * *p* < 0.05; ** *p* < 0.01, Student’s *t*-test.

**Figure 4:**
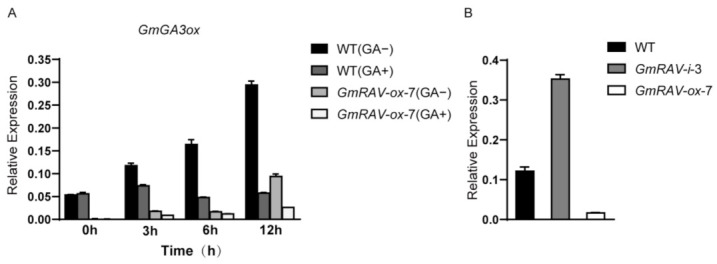
Quantitative real-time PCR analysis of *GmGA3ox.* (**A**) The expression of the *GmGA3ox* gene in soybean leaves treated with GA_3_. The 15-day-old seedlings were sprayed with 100 µM GA_3_, and leaf samples were sampled at 0, 3, 6 and 12 h after treatment. (**B**) The expression of *GmGA3ox* gene in WT, *GmRAV-i-3* and *GmRAV-ox-7* soybean.

To further investigate whether GAs was involved in the restoration of hypocotyl and stem length of *GmRAV-ox* soybean, soybean seeds of wild type and three transgenic *GmRAV-ox* lines were germinated for three days, and the seedlings were then transferred to a fresh medium containing GA_3_ for hypocotyl elongation assay. For the stem elongation assay, three transgenic *GmRAV-ox* lines were sprayed with GA_3_ to determine whether exogenous GA could rescue their shorter height phenotype. In *GmRAV-ox* plants treated with GA_3_, the dwarf phenotypes of the *GmRAV-ox* lines were fully restored to the same hypocotyl length by the supplement of GA_3_ as the wild type seedling without treatment, though the hypocotyl length of the wild type seedlings treated with GA_3_ were more evidently increased (Figure 3A,B). As for the increase in stem length by GA_3_, the final heights of transgenic plants were restored though not as high as wild type after GA_3_ treatment (Figure 3C,D), which suggested that the *GmRAV* gene could be involved in the GA pathway. The detection of the endogenous GA_3_ levels also showed that the endogenous GA_3_ level in *GmRAV-ox* soybean plants was significantly lower than that in WT (Figure 3E). Furthermore, our previous RNA-seq data indicated soybean GA metabolism gene, GA4 biosynthetic genes *GmGA3ox* (*Glyma.07G033800*, *Glyma.13G361700*) were repressed in *GmRAV-ox* soybean 1.75 and 4.56 fold, respectively [19]. The expression of more fold upregulation of *GmGA3ox* (*Glyma.13G361700*) was further analyzed in wild type and *GmRAV-ox-7* seedlings treated with and without 100 µM GA_3_. GA metabolic gene GmGA3ox was subjected to complex regulation based on negative feedback and positive feed-forward mechanisms owing to an excess of GA_3_ spray in both genotypes (Figure 4A). The expression levels of *GmGA3ox* were repressed in both wild type and *GmRAV-ox-7* soybean leaves from 3 to 12 h after GA_3_ treatment. In addition, *GmGA3ox* was significantly downregulated in *GmRAV-ox-7* compared with the wild type (Figure 4A), but the repressing effect of GA_3_ on the expression levels of *GmGA3ox* in the wild type was more significant than those in *GmRAV-ox-7* soybean leaves. Moreover, the expression of *GmGA3ox* was also significantly upregulated in *GmRAV-i-3*, which indicated the repression of *GmRAV* on GA biosynthesis *GmGA3ox* gene (Figure 4B). Together, these findings revealed that the *GmRAV-ox-7* had low active gibberellin levels and *GmRAV* might negatively regulate the GA metabolic gene *GmGA3ox* to limit soybean plant height.

### 2.3. Identification of GmGA3ox as Direct Target of GmRAV

**Figure 5:**
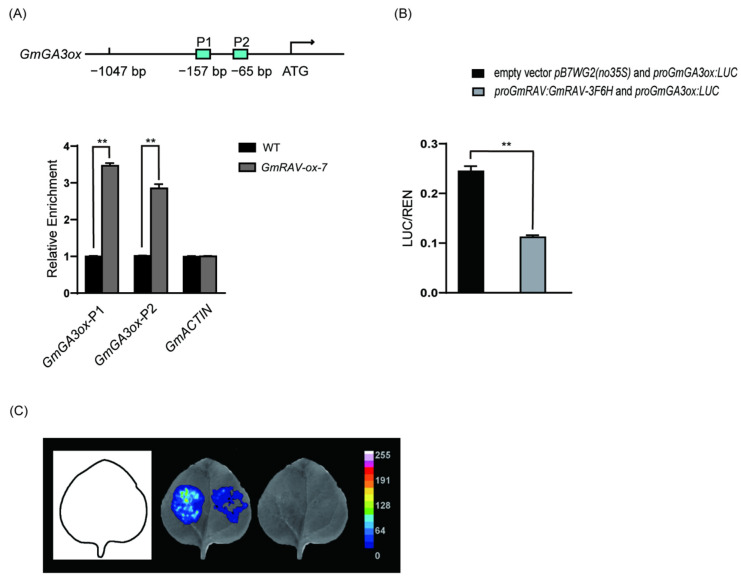
GmRAV physically associated with *GmGA3ox* promoter and repressed its transcription. (**A**) ChIP analysis of *GmRAV* binding to *GmGA3ox* regulatory regions. Precipitated chromatin DNA was used as template in qPCR. Relative enrichment of *GmGA3ox* fragment containing two CAACA motifs marked P1 and P2 indicated two regions from the regulatory region of *GmRAV* up stream of the ATG. The top image shows the locations of the PCR fragments in *GmGA3ox* gene. The *GmACTIN4* locus was used as a negative control. Values are shown as means ± standard deviation (SD) from three biological replicates. Student’s *t*-test, ** *p* < 0.01. Upper panel: physical locations of 1047 bp fragments harboring putative motifs are shown in the schematic diagram. (**B**) Relative luciferase activity was monitored in tobacco leaves co-transfected with effector *proGmRAV:GmRAV-3F6H* or empty vector *pB7WG2 (no35S)* and reporter construct *proGmGA3ox:LUC*. The activities of firefly LUC were normalized by the activities of 35S::Renilla LUC. Student’s *t*-test, ** *p* < 0.01. Results represent means ± SD of six independent samples. (**C**) Luciferase assay of *proGmRAV:GmRAV-3F6H, pB7WG2 (no35S)* and *proGmGA3ox:LUC* constructs at 12 h. D-luciferin was used as the substrate of LUC. Left: *pB7WG2 (no35S) + proGmGA3ox:LUC*; right: *proGmRAV:GmRAV-3F6H + proGmGA3*ox:*LUC*.

As previously reported, GmRAV could directly bind to both CAACA and CACCTG motif by DAP-seq and electrophoretic mobility shift assay (EMSA) [19]. Therefore, *GmGA3ox* were also further analyzed to determine whether *GmRAV* could directly bind them by chromatin immunoprecipitation (ChIP). The two RAV-binding site CAACA motifs (P1 and P2) were located in promoter of *GmGA3ox* in soybean (Figure 5A). ChIP-qPCR was carried out on the leaves of 20-day-old *GmRAV-ox-7* seedlings to verify potential GmRAV-binding sites in promoter of *GmGA3ox* with wild type sample as a negative control. There was significant enrichment in *GmGA3ox* promoter P1 and P2 regions upstream of the ATG by ChIP-qPCR (Figure 5A), indicating that *GmGA3ox* promoter containing two CAACA motifs was bound by GmRAV. Moreover, the expression levels of *GmGA3ox* in leaves were both elevated in *GmRAV-i-3* and decreased in *GmRAV-ox-7* soybean plants (Figure 4B).

We also tested the functional interaction of GmRAV on CAACA in *GmGA3ox* promoter in vivo by using a transient expression assay in *N. benthamiana* (Figure 5B). In this system, the LUC reporter gene was driven by *GmGA3ox* promoter containing two CAACA motifs. When co-infiltrating *Agrobacterium* expressed *proGmRAV:GmRAV-3F6H* (3 × FLAG and 6 × Histidine) effectors together with the *proGmGA3ox:LUC* reporter into *N. benthamiana* leaves, the activity of LUC specially decreased (Figure 5B,C), thus demonstrating that *GmRAV* could inhibit the transcriptional activation activities of *GmGA3ox*. Together, our results suggested that GmRAV protein repressed the expression of *GmGA3ox* by directly binding to the CAACA motif in the promoter.

## 3. Discussion

In plants, changes in a single gene could enhance the improvement of multiple important agronomic traits [19,22,23,24]. For example, the loss-of-function *J*-alleles not only delayed soybean maturity and enhanced grain yield in soybean, but also enhanced tolerance to salt stress [22]. The suppression of *MicroRNA168* (*miR168*), which encodes a key component of the RNA-induced silencing complex, not only improved grain yield and shortened flowering time in rice, but also enhanced immunity to *M.oryzae* [23]. Previously, we reported that three *GmRAV-ox* transgenic soybean lines displayed flowering time compared with non-transgenic soybean lines, and *GmRAV-RNAi* transgenic lines showed earlier flowering time [19]. In this study, plant height phenotypes in *GmRAV-ox* and *GmRAV-RNAi* transgenic soybean plants were further observed. Compared with the wild type soybean plants control, *GmRAV-ox* plants displayed decreased height. Conversely, *GmRAV-RNAi* transgenic soybean plants displayed significantly increased height. These results clearly showed that the alteration of *GmRAV* amounts affected plant height and flowering time and might influence yield.

Plant height is a key parameter that describes plant growth status for breeding in various crops [3,4,25]. GAs is a key plant hormone that regulates diverse biological processes throughout the life cycle of plants, such as embryogenesis, leaf primordia, flowering, plant height and developing anthers [5,26,27]. We thus detected the levels of endogenous GA_3_ in *GmRAV-ox* soybean plants, and the results show that the endogenous GA_3_ level in *GmRAV-ox* soybean plants was significantly lower than that in WT, and the shortened internode phenotype could be rescued by treatment with exogenous GA_3_. In recent years, some GA-metabolic-pathway-related genes associated with plant height have been reported in plants [8,28]. For example, a loss function of *GA3ox* gene led to GA4 reduction and dwarfism phenotype in watermelon [29]. To further analyze the mechanism of *GmRAV* regulating plant height, based on our previous RNA-seq data that GA4 biosynthetic genes *GmGA3ox* were repressed in *GmRAV-ox* soybean [19], the expression levels of the *GmGA3ox* in the *GmRAV-ox* and *GmRAV-RNAi* transgenic soybean plants were further tested. We found that *GmGA3ox* gene had substantially decreased expression in the *GmRAV-ox* transgenic soybean plants and increased expression in *GmRAV-RNAi* transgenic soybean plants. Overall, we speculated that *GmRAV* might negatively regulate the expression of these GA-biosynthesis-pathway-related genes to limit soybean plant height.

The RAV transcription factors played important roles in flowering time, heading date and stress responses, which they mediated by binding to the CAACA and CACCTG sequences in the promoters of target genes [15,16,19]. In soybean, two GmRAV binding motifs [C(A/G)AACAA(G/T)A(C/T)A(G/T)] and [C(T/A)A(C)C(T/G)CTG] were identified in our previous reported [19]. In the current study, we used ChIP-qPCR analysis, and demonstrated that GmRAV directly bound to the CAACA motif of *GA3ox* gene promoters. The results above together suggested that GmRAV directly repressed *GA3ox* gene expression by binding to two CAACA motifs in their promoters. However, in *Arabidopsis thaliana*, *TEM* genes that also belonged to RAV family directly repressed the expression of the *GA3OX1* and *GA3OX2* by directly binding a regulatory region positioned in the first exon. Plants overexpressing *TEM* genes resembled GA-deficient mutants as a result of an decrease in GA content [30].

Based on our data, we proposed a model of GmRAV as a negative regulator regulating the plant height in soybean by binding to the promoter of *GA3ox* gene, directly repressing their expression. Subsequently, the reduced expression of *GA3ox* gene led to decreased endogenous GA_3_ levels and plant height. Our findings provided a new insight into the mechanisms underlying plant height regulatory networks in soybean and offered a strategy for breeding plant ideotypes by genetically manipulating a *GmRAV* gene.

## 4. Materials and Methods

### 4.1. Plant Materials, Growth Conditions and Records of Data

For statistical experiment of soybean phenotype and agronomic traits, T_7_ generation *GmRAV-i-3*, three T_4_ generation *GmRAV-ox-5*, *GmRAV-ox-7* and *GmRAV-ox-14* lines [19] and WT soybean seeds were planted in pots in the field under natural light conditions in May in Harbin. At least 15 plants were analyzed each line each time, and the experiments were repeated 3 times. Means ± SD deviation was used in the statistical analysis of the data. To analyze the response of *GmGA3ox* to GA_3_, 15-day-old soybean ‘Dongnong 50′ seedlings planted at 25 °C, 250 µmol m^−2^ s^−1^ white light, 16/8 h light/dark condition were sprayed with 100 µM GA_3_, and trifoliate leaves were sampled at 0, 3, 6 and 12 h after treatment.

### 4.2. Plasmids Construction

To generate *GmGA3ox* promoter-driven *LUC* constructs *proGmGA3ox:LUC*, the promoter DNA was amplified from genomic DNA of ‘Dongnong 50′ by using *pro GmGA3ox:LUC*-F and *proGmGA3ox:LUC*-R primers (Table A1). The PCR products were purified and cloned into binary vector pGreenII-0800-LUC linearized by *Sma*I using In-Fusion cloning system (TaKaRa, Tokyo, Japan), respectively. The recombinant constructs were introduced into *Agrobacterium GV3101* and subsequently transformed into *N. benthamiana* [31].

### 4.3. Quantitative Real-Time RT-PCR

Total RNA was extracted from soybean leaves with RNAiso Plus Kit (TaKaRa, Tokyo, Japan), and then was reverse-transcribed into first-strand cDNA in a 20 µL volume with PrimeScript RT reagent Kit (TaKaRa, Tokyo, Japan). The expression of *GmGA3ox* was analyzed by quantitative real-time RT-PCR (qRT-PCR). QRT-PCR amplifications were performed [32]. The PCR cycling conditions: 94 °C for 30 s; 40 cycles of 94 °C for 5 s, 60 °C for 30 s. *GmActin4* (GenBank accession number AF049106) were used as reference gene. The primers used in qRT-PCR analyses were shown in Table A1. Three biological replicates and three technical replicates were applied for all experiments.

### 4.4. ChIP-qPCR

Wild type and soybean transgenic *proGmRAV:GmRAV-3F6H-ox-7* lines were grown for 20 days under natural light conditions. Approximately 1 g of trifoliate leaves from wild type and *GmRAV-ox-7* transgenic line were harvested at ZT 12 h, fixed, and quenched by glycine. Nuclei were isolated and lysed, and the chromatin solution was then sonicated to approximately 200–1000 bp DNA fragments. Immunoprecipitation reactions were performed by using anti-FLAG antibody and anti-IgG antibody control [19]. The enrichment of DNA sequence segments in *GmGA3ox* promoter was chosen to perform qPCR. Three biological repeats and three technical replicates were performed for each sequence segment. *GmACTIN* was used as the internal gene control. The primer pairs used in ChIP-qPCR were listed in (Table A1).

### 4.5. Transient Assay of GmGA3ox Promoter Affected by GmRAV Protein in N. benthamiana

The constructs *proGmRAV:GmRAV-3F6H* [19] and *proGmGA3ox:LUC* were simultaneously transferred into *N. benthamiana* to measure transient assay of *GmGA3ox* promoter affected by *GmRAV* protein. Three independent experiments were performed and each experiment was repeated three times to obtain reproducible results. The luminescence signal was captured using Amersham Imager 600 (GE Healthcare, Amersham, UK) after spraying 1 mM luciferin on *N. benthamiana* leaves. The transient activity of recombinant vector *proGmGA3ox:LUC* was assayed by using dual luciferase assay kit (Promega, Madison, WI, USA) and multifunctional microplate reader TECAN Infinite 200 PRO (Tecan Schweiz, Männedorf, Switzerland) [19].

### 4.6. Hypocotyl and Stem Growth Assay of Seedlings

The seeds of *GmRAV-ox-5*, *GmRAV-ox-7* and *GmRAV-ox-14* and WT were used to test the restoration of GA-mediated hypocotyl elongation. After normal germination on MS medium for 3 days, all soybean seeds were transferred to MS medium containing 0 and 10 µM GA_3_, respectively [32]. For the stem elongation assay, three transgenic *GmRAV-ox* lines were sprayed with 100 µM GA_3_.

### 4.7. Scanning Electron Microscopy

The internode cells of *GmRAV-ox-7* and WT soybeans were observed using an S-3400N scanning electron microscope (Hitachi Ltd., Tokyo, Japan) equipped with a cooling table [32].

### 4.8. Endogenous GA_3_ Determination

A total of 1 g leaves were harvested from 20-day-old *GmRAV-ox-5*, *GmRAV-ox-7*, *GmRAV-ox-14* and WT soybean seedlings. Plant GA_3_ ELISA Kit (Andy gene) was used to determine the endogenous GA_3_ levels in three transgenic lines and WT plants. The absorbance (OD) of the samples was measured at 450 nm with a microplate analyzer. The concentration of GA_3_ in the samples was calculated by the standard curve [32].

## 5. Conclusions

In conclusion, GmRAV protein repressed the expression of *GmGA3ox* by directly binding to the two CAACA motifs in the promoter to limit soybean plant height. GmRAV was involved in the regulation of plant height directly by mediating key components of the GA synthesis pathway.

## Data Availability

Sequence data from this article can be found in the Phytozome database under the following accession numbers: *GmRAV(Glyma.10G204400), GmGA3ox(Glyma.13G361700).*

## Appendix A

**Table A1:** The specific sequences of the primers.

Primer	Sequence (5′-3′)
*qGmGA3ox 1-ChIP-F*	TCTCCCTCAACTTTCAGTTCCA
*qGmGA3ox 1-ChIP-R*	TCAATTCTCCCTTTCAGCCTTC
*qGmGA3ox 2-ChIP-F*	TCCTGAAGGCTGAAAGGGAGAA
*qGmGA3ox 2-ChIP-R*	ACCGCATGGAAATTAGATAGAG
*qGmGA3ox-F*	AGCCATGATGATCATACTCCTG
*qGmGA3ox-R*	CAAGTTATGCATGCATGGTGTA
*proGmGA3ox: LUC-F*	GAATTCCTGCAGCCCAACCGATTCAAATTGAGGGTAT
*proGmGA3ox: LUC-R*	ACTAGTGGATCCCCCACCGCATGGAAATTAGATAGAG
*qGmACTIN4-F*	GTGTCAGCCATACTGTCCCCATTT
*qGmACTIN4-R*	GTTTCAAGCTCTTGCTCGTAATCA
